# Expediting Glycospace Exploration: Therapeutic Glycans via Automated Synthesis

**DOI:** 10.1002/anie.202422766

**Published:** 2025-02-21

**Authors:** James Suri, Ryan Gilmour

**Affiliations:** ^1^ Institute for Organic Chemistry University of Münster Corrensstraße 36 48149 Münster Germany; ^2^ Cells in Motion (CiM) Interfaculty Center Röntgenstraße 16 D-48149 Münster Germany

**Keywords:** automation, carbohydrates, glycans, glycospace, therapeutics

## Abstract

Glycans regulate a vast spectrum of disease‐related processes, yet effectively leveraging these important mediators in a therapeutic context remains a frontier in contemporary medicine. Unlike many other classes of clinically important biopolymers, carbohydrates derive from discrete biosynthetic pathways and are not produced directly from genes. The conspicuous absence of a biological blueprint to achieve amplification creates a persistent challenge in obtaining well‐defined glycostructures for therapeutic translation. Isolating purified sugars from biological sources is not without challenge, rendering synthetic organic chemistry the nexus of this advancing field. Chemical synthesis has proven to be an unfaltering pillar in the production of complex glycans, but laborious syntheses coupled with purification challenges frequently introduce reproducibility issues. In an effort to reconcile these preparative challenges with the societal importance of glycans, automated glycan synthesis was conceptualised at the start of the 21st century. This rapidly expanding, multifaceted field of scientific endeavor has effectively merged synthetic chemistry with technology and engineering to expedite the precision synthesis of target glycans. This minireview describes the structural diversity and function of glycans generated by automated glycan synthesis platforms over the last five years. The translational impact of these advances is discussed together with current limitations and future directions.

## Graphical abstract

The construction of well‐defined carbohydrates constitutes a nexus in glycoscience, but few conceptual paradigms have shaped the field as much as automated glycan synthesis. This minireview describes the structural diversity and function of glycans generated by automated glycan synthesis platforms over the last five years. The translational impact of these advances, their limitations and future perspective are discussed.

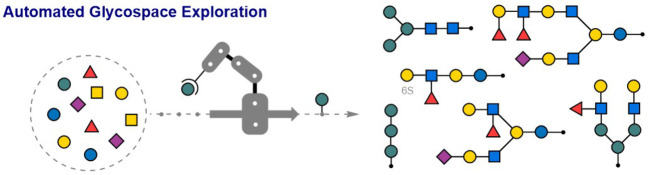

## 1 Introduction

**Figure 1:**
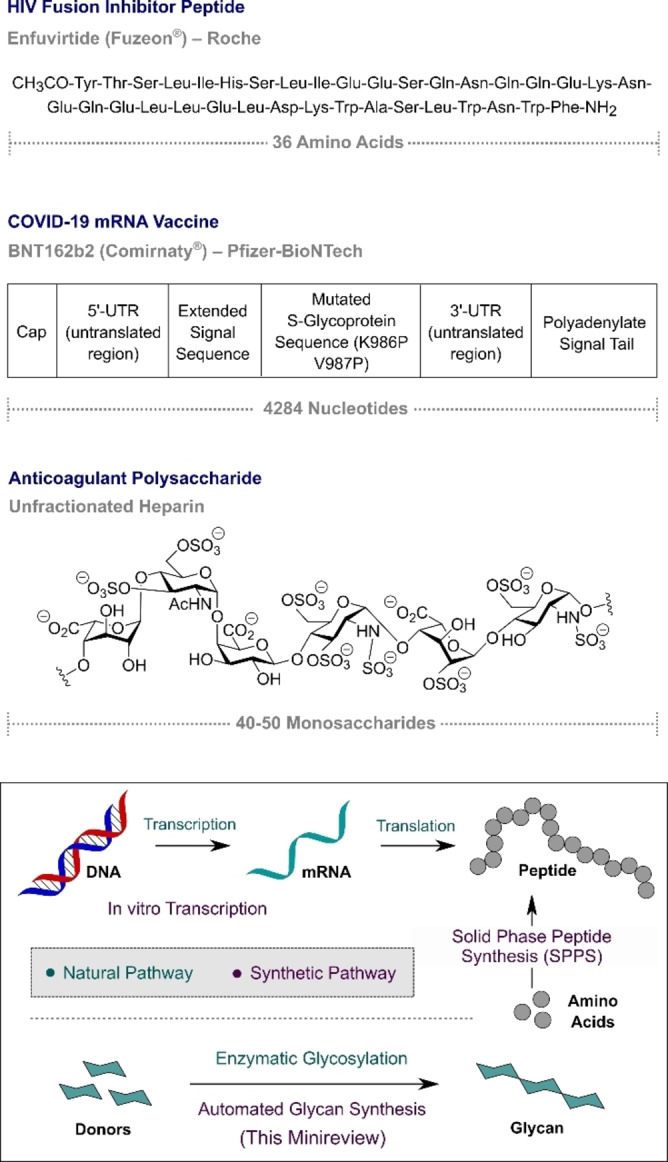
Examples of therapeutic biopolymers (peptides, mRNA and glycans).

The centrality of glycans in human physiology and medicine is manifest in the eclectic spectrum of biological processes which they regulate.^[1]^ In this multiscale enterprise, the abundance of carbohydrates in metabolism (glycolysis) contrasts starkly with the fleeting concentrations of glycans that act as essential diagnostic markers of inflammation and other disease pathologies.[^2^, ^3^, ^4^, ^5^, ^6^] The complexity and diversity of glycans therefore provide a remarkable degree of structural latitude such that glycan heterogeneity constitutes the basis of a molecular fingerprint system:[^7^, ^8^] this is an appealing prospect from the perspective of personalised medicine and drug discovery.^[9]^ Despite the success and widespread use of many carbohydrate‐based drugs,[^10^, ^11^, ^12^, ^13^, ^14^] the ubiquity of glycans in biology has thus far not translated to equal prominence in the clinic. Although this is reflective of a multifaceted problem that includes glycan stability in vivo[^15^, ^16^] and weak interactions with target proteins,^[17]^ challenges associated with construction continue from the nexus of this discussion.[^18^, ^19^] Echoing the *“3Cs Model”*, the successful construction of complex glycans is subject to alliteration: constitution, configuration and conformation must be simultaneously addressed.[^20^, ^21^] This distinguishes glycans from peptide‐ and nucleic acid‐based biopolymers, which can be amplified using bioinspired, linear coupling strategies.^[22]^ The advent and development of solid phase peptide synthesis (SPPS)^[23]^ and in vitro transcription (IVT) paradigms to secure reliable access to mRNA,^[24]^ has revolutionised the synthesis of these two biomolecule classes. The existing disparity in biomolecule synthesis is immediately evident upon inspection of commonly used synthetic therapeutic biopolymers (Figure 1). In the arena of synthetic peptides, the HIV fusion inhibitor enfuvirtide (Fuzeon®), contains an impressive 36 amino acids.^[25]^ The COVID‐19 mRNA vaccine BNT162b2 (Comirnaty^®^) is composed of a staggering 4284 nucleotides.^[26]^ In contrast, one of the larger polysaccharide therapeutics on the market is the anticoagulant, unfractionated heparin.^[27]^ The composition ranges from 40–50 monosaccharides, but the molecule is not produced synthetically and is instead isolated from animal (e.g. bovine) sources.^[28]^ Whilst this comparison illustrates the complexity of biopolymers in clinical medicine,[^29^, ^30^] it is important to emphasise the potential of carbohydrates in fields such as immunology where glycan‐based vaccines are well‐represented.[^31^, ^32^, ^33^] Often, heterogeneous mixtures isolated from natural sources are frequently employed in the absence of effective, robust and economical synthetic approaches.^[34]^ This reliance on glycans isolated from biological sources, often as mixtures, has led to concerns regarding the reproducibility of the supply chain and presents barriers in establishing structure–activity relationships as a part of sustained medicinal chemistry campaigns.[^35^, ^36^] Consequently, efforts to devise robust platforms to access libraries of well‐defined glycans in sufficient amounts for biological investigations is a burgeoning field of research motivated by the societal implications in translational neurology,^[37]^ oncology^[38]^ and virology.^[39]^

In the absence of amplification strategies, access to complex glycans has traditionally been achieved through chemical synthesis campaigns that often suffer from lengthy sequences and irreproducibility; this renders translation to commercial manufacturing challenging.^[40]^ To combat these shortcomings, automated glycan synthesis was conceptualised to expedite reproducible access to complex glycans and meet the demand created by advances in glycobiology.^[41]^ The rapid and successful expansion of this paradigm is evident from the compelling reports of a semisynthetic *S. pneumoniae* vaccine candidate,^[40]^ unnatural hairpin structures,^[42]^ and a polyarabinoside 1080‐mer,^[43]^ to name only three examples. In this minireview, the rapid growth and impact of therapeutic glycan synthesis via automated approaches over the last five years will be surveyed. In this short time period, the explosive growth in the automated precision synthesis of target drugs, vaccine leads and biological standards suggests a conceptual shift in the generation of glycans to investigate physiological processes and guide drug development.

## 2 Automated Glycan Synthesis

**Figure 2:**
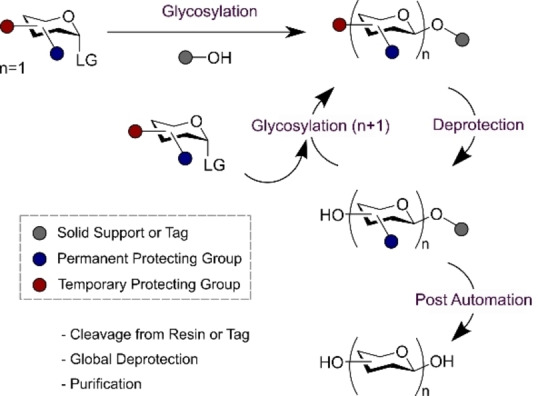
Overview of automated glycan synthesis including the key steps of glycosylation and deprotection.

Automated carbohydrate synthesis continues to gain traction, providing momentum for the development of diverse conceptual approaches to enable this objective. These advances have been the subject of a number of excellent reviews on the topic,[^44^, ^45^, ^46^, ^47^, ^48^] which include detailed technical discussions on solid‐ and solution‐phase glycosylations enabled by chemical, photochemical, enzymatic and electrochemical activation. Many of these approaches share a common workflow blueprint that consists of repeated cycles of glycosylation and deprotection of a temporary protecting group followed by post automation cleavage from the support (for solid phase) or from a tag (solution phase). Global deprotection and purification completes the synthetic sequence (Figure 2). It is important to note that enzymatic platforms are conceptually distinct on account of the fact that the donors do not carry protecting groups.

Automated solid‐phase oligosaccharide synthesis was first achieved by Seeberger and co‐workers in 2001 using a modified peptide synthesiser.^[49]^ By 2012, the same group had designed the first dedicated solid phase automation instrument^[50]^ with the first commercially available synthesiser following: the Glyconeer 2.1®.[^51^, ^52^] The conceptual framework of this strategy centres upon a reaction chamber containing resin‐bound acceptor; this is successively filled and drained of reagents and donors. Standard cycles include glycosylation, deprotection and capping, with intermediate washing steps integrated to remove excess reagents. Photolabile linkers are commonly employed to simplify the cleavage of the glycan from the resin prior to purification and global deprotection.^[53]^

In 2012, Demchenko and co‐workers described HPLC‐assisted automation (requiring minor operator input) utilising resin‐bound acceptor localised in a column, through which reagents and donors are washed.^[54]^ The same group released an updated version in 2020 (Gen C HPLC‐A) that was fully automated.^[55]^ Solution phase HPLC‐A was reported in 2022; this system is capable of running multiple glycosylations in a batch format.^[56]^ Fluorous tags, as handles for purification, formed the basis of the solution phase automation platform introduced by Pohl in 2015.^[57]^ Following cycles of glycosylation and deprotection, fluorous solid phase extraction enables the glycosylation product to be purified and then returned to the reaction vessel for subsequent coupling events. In 2013, Nokami, Yoshida and co‐workers devised a platform for solution phase electrochemical automation in which anodic oxidation generates a reactive intermediate triflate from the corresponding thiodonor, to which the glycosyl acceptor is added.^[58]^ Multiplicative solution phase automation was introduced by Ye and co‐workers in 2022, where the approach is predicated on pre‐activation of the donor, either chemically or photochemically.^[43]^ The multiplicative nature of this strategy lends itself to the construction of extremely large glycans, and the authors convincingly validated this by generating a 1080‐mer.

To complement chemical glycosylation platforms, Boons and co‐workers disclosed an automated enzymatic glycan synthesiser in 2019.^[59]^ The strategic introduction of sulfonate tags at the reducing end of the growing glycan chain allows temporary trapping of the glycan in an ion exchange column to aid intermediate purification. Mildly acidic conditions enable the tag to be removed and yield the glycan with a free reducing end. The enzymatic nature of this method mitigates the requirement for protecting groups on the donor. A complementary enzymatic platform was developed by Elling, Franzreb and co‐workers that leverages automated enzyme cascades using an immobilised microfluidic enzyme reactor.[^60^, ^61^] The growing glycan passes through sequential compartments, each containing enzymes immobilised on magnetic beads, to facilitate glycosylation. It is pertinent to note that Wang, Wen and co‐workers disclosed the semi‐automated synthesis of glycopeptides using a peptide synthesiser in 2020.^[62]^ The peptide is constructed using SPPS, with the inclusion of an unnatural amino acid building block bearing an unprotected sugar that is then extended enzymatically using UDP‐donors. The method was termed semi‐automated due to the requirement for a switch from organic solvents for the SPPS to water for the enzymatic glycan extension.

Though the following methods were not employed in the examples described in this minireview, they represented significant advances in the field and are therefore it is pertinent to highlight them at this juncture. In 2010, Nishimura and co‐workers developed a HPLC‐based enzymatic platform that utilised soluble dendrimers as a support for the growing glycan chain via a linker.^[63]^ The strategic introduction of a peptide in the linker facilitated facile cleavage with a protease to release the desired glycan. By adapting a peptide synthesiser in 2019, Wen, Wang and co‐workers validated an enzymatic automation platform that employed a thermosensitive polymer support.^[64]^ Although the polymer was soluble at the reaction temperature, heating led to precipitation thereby facilitating separatrion by filtration. It is also important to highlight the contributions of Wong and co‐workers to automating and streamlining the process of building block selection for one‐pot glycosylations through their Optimer^[65]^ and Auto‐CHO^[66]^ programs based on relative reactivity values. This field has been enriched by the more recent “GlycoComputer” programme from Wong, Wang and co‐workers to predict glycosylation outcomes.^[67]^

Although far from comprehensive, these examples serve as important milestones in this evolving area of contemporary biomolecule synthesis and constitute the platform for therapeutic glycan construction.

Though there are many nuances between the methods described above, they can be classified into three broad groups: enzymatic, chemical solid phase, and chemical solution phase. Both chemical solid and solution phase methods utilise protected donor building blocks; the protecting groups are often tailored to control the diastereoselectivity. This facilitates the use of a wide range of possible donors but has the disadvantage that a global deprotection must be performed at the end of the synthesis, which can prove challenging.^[68]^ It is also important to note that the constituent building blocks are often used in excess; therefore, the development of scalable routes to facilitate their synthesis must also be considered. The major benefits of enzymatic methods include the mild aqueous conditions and the use of unprotected donors, thereby mitigating the need for a global deprotection. The selectivity of enzymatic glycosylations (diastereoselectivity and regioselectivity) is often remarkable; however, enzyme specificity often limits the use of modified or unnatural sugars as substrates. Importantly, approaches such as directed evolution have the potential to substantially broaden the use of glycosyltransferase enzymes in synthesis.^[69]^

## 3 Glycosphingolipids

**Figure 3:**
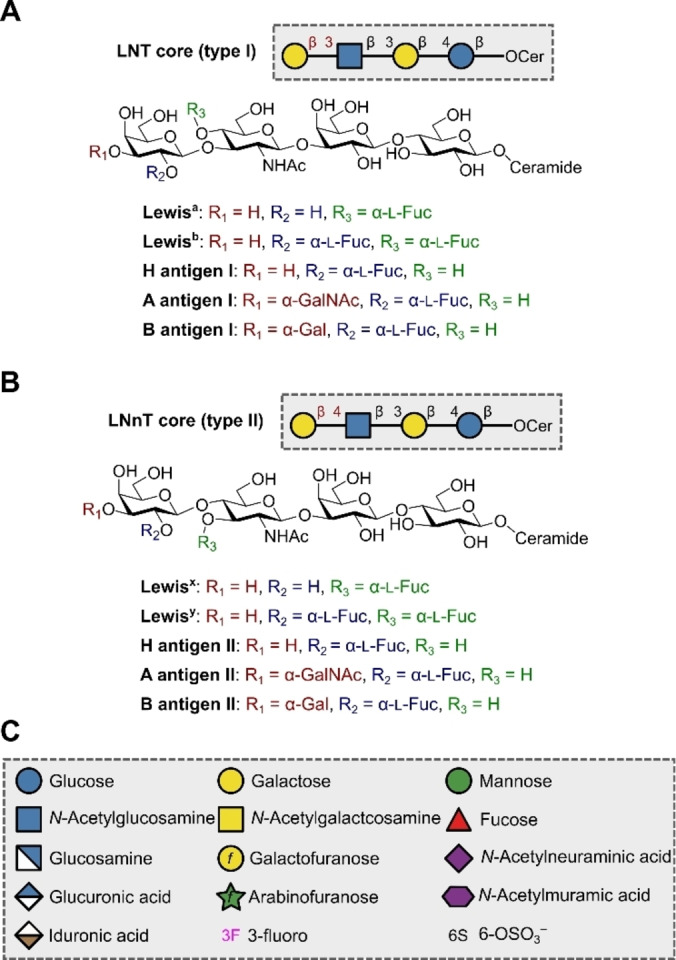
A) LNT core glycosphingolipids. B) LNnT core glycosphingolipids. C) Key for glycan symbol nomenclature used throughout this review.

Glycosphingolipids are a class of glycosylated membrane lipids that display significant structural diversity in both the glycan and the ceramide constituents. There are four common core tetrasaccharide structures, namely the lacto‐series (LNT, type I) (Figure 3A), the neolacto‐series (LNnT, type II) (Figure 3B), the ganglio‐series (Figure 5A) and the globo‐series (Figure 5B): importantly, these motifs share a common lactose motif connected directly to the ceramide portion.^[70]^ These structures are typically sialylated and fucosylated to generate specific glycosphingolipids. Prominent members of the lacto‐ and neolacto‐series are the Lewis antigens (Figure 3A, 3B), which are expressed throughout the body on different cell types. Importantly, the diagnostic overexpression of certain Lewis antigens, e.g. Lewis^x^ and Lewis^x^‐Lewis^y^ (KH‐1), on tumour cells makes these tumour‐associated cancer antigens (TACAs) attractive targets as cancer vaccine leads.^[38]^ The related ABH antigens correspond to the ABO blood groups and are presented on erythrocytes and other epithelial linings as glycolipids and glycoproteins.^[71]^ These antigens are involved in a plenum of host‐pathogen interactions, with a compelling example being the tendency of the bacterium *Helicobacter pylori* to bind the H type I antigen.^[72]^ In the course of developing vaccines or probing pathogen binding, the ability to access well‐defined glycans is essential and is a task for which automation is ideally suited.^[73]^ Solid phase automation with just six building blocks has enabled the synthesis of a panel of Lewis and related antigens: Lewis^a^, Lewis^b^, Lewis^x^, Lewis^y^, Lewis^x^‐Lewis^x^ dimer, KH‐1 (Lewis^x^‐Lewis^y^ heterodimer) and H type II by Seeberger and co‐workers.^[74]^ Microwave‐assisted automation facilitated the rapid synthesis of protected Lewis^x^ in just five hours.^[75]^ Interestingly, by replacing the lipid tail with an aminopentyl linker, a handle for facile conjugation to a microarray is provided. This strategy has proven to be extremely valuable in examining the binding of solid phase automation‐derived Lewis antigens to dendritic cell‐specific intercellular adhesion molecule‐3‐grabbing non‐integrin (DC‐SIGN).^[76]^ Multiplicative solution‐phase automation has also been leveraged recently, by Ye and co‐workers, to furnish protected precursors of Lewis^x^, Lewis^y^, LNT, LNnT, H type I, H type II, A antigen (type II) and B antigen (type II), with impressive yields ranging from 59 % to 85 %.^[43]^

In the case of Lewis antigens, contracted analogues are valuable chemical biology tools, since truncated Lewis antigens found on tumour cells make attractive vaccine targets.^[77]^ Such contracted antigens are not just relevant to oncology research, but to a variety of other cellular processes. L‐selectin is involved in a multitude of physiological processes including lymphocyte homing, inflammatory leukocyte trafficking and demyelination, to name but a few.^[78]^ Understanding how glycan structures influence binding is crucial to elucidating the underlying mechanisms that regulate function and potentially uncovering previously unknown roles. To that end, automation serves an important role.

**Figure 4:**
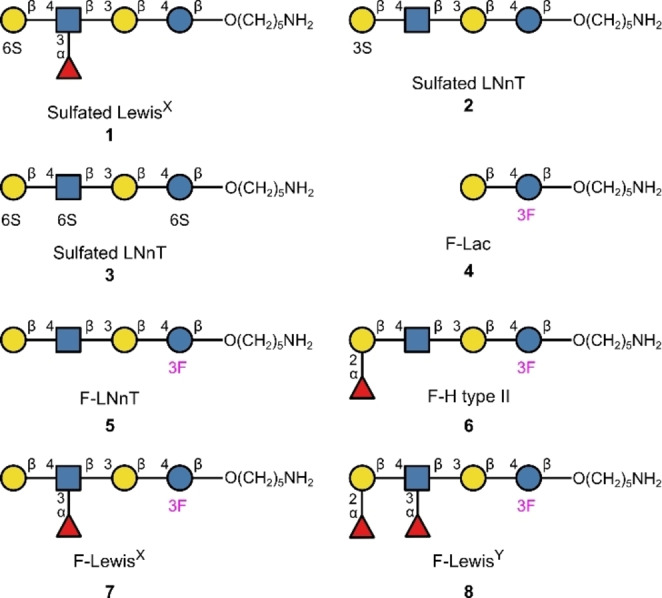
Sulfated or fluorinated glycosphingolipid analogues made using automated glycan synthesis.

In 2022, Delbianco and co‐workers reported a synthesis of sulfated Lewis^x^ **1** (Figure 4), in which the backbone was prepared by solid phase automation followed by on‐resin sulfation.^[79]^ It is known that sulfation can improve the binding of sialyl Lewis^x^ to L‐selectin.^[78]^ However, it has also been observed that sulfation can substitute sialylation in Lewis^x^ and that further investigation of these interactions is required.[^80^, ^81^] In the same study, the syntheses of two sulfated LNnT derivatives (**2** and **3**) were also disclosed and these compounds may be valuable in investigating binding to human galectin‐4; this has relevance in cancer research.^[82]^

The importance of glycan binding has led to the development of a powerful suite of methods to probe these phenomena.^[83]^ ^19^F NMR has emerged as a particularly efficient and operationally simple method to investigate the binding of selectively fluorinated carbohydrates to proteins.^[84]^ This approach is appealing on account of the ability of fluorine to be leveraged to mitigate hydrolysis and regulate glycosylation selectivity, if appropriately positioned.[^15^, ^16^, ^84^, ^85^] Motivated by the many appealing attributes of fluorinated carbohydrates, solid phase automation has been leveraged, by Rademacher, Delbianco and co‐workers, to generate fluorinated Lac **4**, LNnT **5**, H type II **6**, Lewis^x^ **7** and Lewis^y^ **8** to probe glycan‐protein interactions using ^19^F NMR .^[86]^ These di‐ to hexasaccharides were produced in yields of 5–20 %, and the fluorine was remotely located at the 3‐position of the lactose glucose, to minimise any impact on binding. Through ^19^F NMR‐enabled binding analysis, the authors confirmed known binding trends to seven proteins, including DC‐SIGN (involved in HIV‐1 infection) and BambL (involved in lung infections), thus validating their methodology. Furthermore, the ^19^F‐reporter allowed enzymatic reactions of the synthesised glycans to be followed in real time. Collectively, these findings constitute an expedited process for probing protein‐glycan binding and monitoring enzymatic reactions by combining automated synthesis with ^19^F NMR studies.

**Figure 5:**
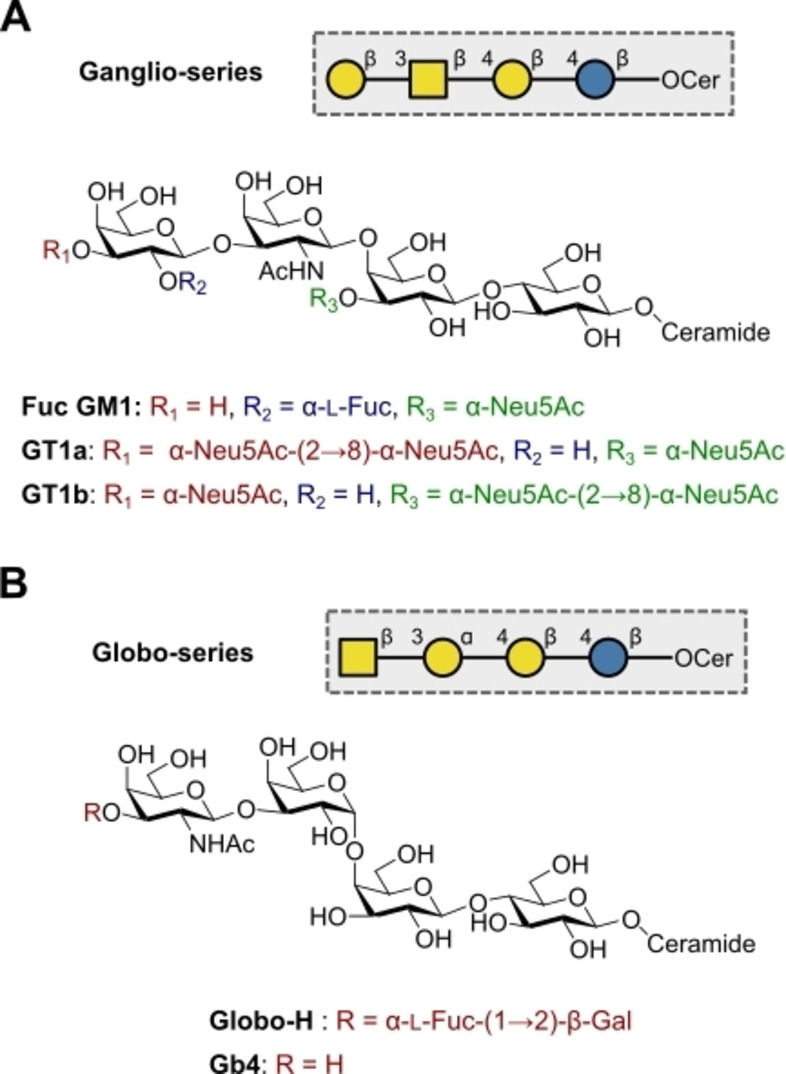
A) Ganglio‐series glycosphingolipids. B) Globo‐series glycosphingolipids.

Gangliosides are a structurally related class of glycosphingolipids that are widely expressed on cell surfaces; certain motifs are TACAs, of which Fuc‐GM1 is an example in small‐cell lung carcinoma (Figure 5A).[^87^, ^88^] In 2022, Ye and co‐workers used solution phase multiplicative automation to produce protected Fuc‐GM1 in 51 % yield using a [1+2+3] strategy.^[43]^ The versatility of this strategy also enabled the trimer component to be forged in an automated fashion from lactose and Neu5Ac.

Cell differentiation is a key process in cancer progression, which is regulated by epidermal growth factor (EGF) among others. EGF binds to the epidermal growth factor receptor (EGFR), inducing auto‐phosphorylation to create binding sites for signalling molecules that stimulate proliferation.^[89]^ Gangliosides are known inhibitors of this auto‐phosphorylation process and are therefore of interest in oncology research.[^90^, ^91^] In a study of a small ganglioside class, GT1b was found to be the most potent inhibitor of EGFR phosphorylation in the human neuroblastoma cell line, NBL‐W.^[92]^ The authors postulated that the number and arrangement of Neu5Ac residues were a key contributor to the inhibitory potency. In the context of this review, the enzymatic automation of GT1b and the related GT1a scaffold (Figure 5A), by Boons and co‐workers in 2019, is particularly relevant.^[59]^ This investigation convincingly demonstrates how effective automation is in the production of libraries of gangliosides to define precise structure–activity relationships. Starting from lactose, GT1a and GT1b were prepared over five cycles in 18 % and 24 % yields, respectively. Furthermore, GT1a is a disease‐relevant ganglioside in its own right, as anti‐GT1a IgG is implicated in Guillain‐Barré syndrome (GBS).^[93]^ GBS is a peripheral neuropathy that usually follows acute infection, from *Campylobacter jejuni* for example, which can mimic GT1a with its surface lipooligosaccharides (LPS).^[94]^ The resulting autoantibodies then attack components of the nervous system presenting GT1a, resulting in neurological symptoms.^[95]^

Globo‐H (Figure 5B), a globoside TACA first isolated on MCF7 breast cancer cells, has long been investigated as a cancer vaccine.^[96]^ Globo‐H has a venerable history in automated glycan synthesis following Seeberger's report.^[97]^ This has recently been complemented by a multiplicative solution phase automation approach to yield the protected Globo‐H hexasaccharide in 49 % yield from a [1+2+1+2] coupling.^[43]^ With the objective of targeting TACAs using nanobodies (single domain antibodies), Seeberger, Moscovitz and co‐workers immunised an alpaca with different glycoconjugates, with Globo‐H among the epitopes tested.^[98]^ The isolated nanobody with the highest binding to MCF7 cells was characterised and it was determined that this nanobody was specific enough that it did not bind HEK293 or MCF10A; these cells express Gb2 (α‐Gal(1→4)‐β‐Gal‐Cer), Gb3 (α‐Gal‐(1→4)‐β‐Gal‐(1→4)‐β‐Glc‐Cer) and Gb4 (Figure 5B), substructures of Globo‐H. Highly specific nanobodies targeting TACAs are evidently of great interest and this work illustrates the impact of automation in facilitating nanobody exploration.

## 4 Human Milk Oligosaccharides

**Figure 6:**
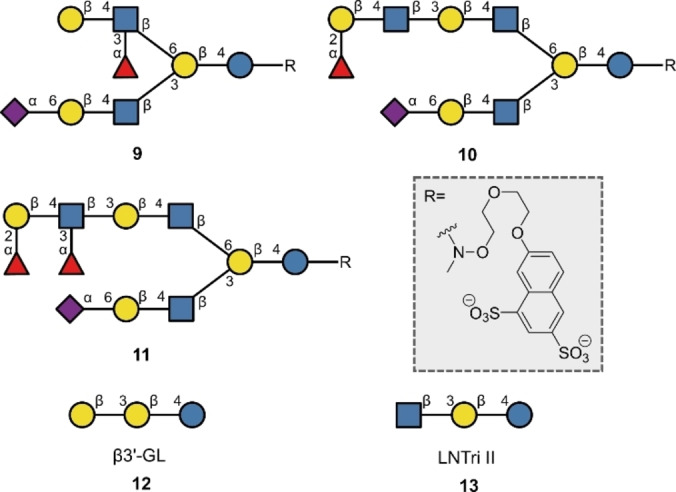
A) Human milk oligosaccharides made using automated glycan synthesis.

Human milk oligosaccharides (HMOs) are a group of glycans found (as the name suggests) in human milk and consist of combinations of five monosaccharides: Glc, Gal, GlcNAc, Fuc and Sia (Neu5Ac in humans) with a conserved lactose reducing end moiety (Figure 6).[^99^, ^100^] HMOs are well known as prebiotics, such that beneficial or “*good*” bacteria can effectively utilise HMOs for growth.[^101^, ^102^] However, these glycans also offer protection by possessing similar epitopes to those targeted by pathogen lectins. Hence, unconjugated HMOs can sacrificially bind the pathogens to prevent them from interacting with their intended, cell‐bound targets that facilitate infection.^[103]^ Furthermore, Lewis antigen‐containing HMOs can bind host DC‐SIGN to prevent gp120‐mediated HIV binding.^[104]^ HMOs can also act as antimicrobials and have been found to increase the membrane permeability of some bacteria.^[105]^ Although the mechanisms by which these varied glycans interact with other biological processes have gained clarity, progress in this area continues to be compromised by difficulties in accessing pure glycan samples.^[106]^

In 2019, Boons and co‐workers harnessed enzymatic automation to produce three HMOs: **9** (containing Lewis^x^), **10** (containing H type II**)** and **11** (containing Lewis^y^) in 37 % (6 enzymatic steps), 21 % (8 enzymatic steps) and 16 % (9 enzymatic steps), respectively (Figure 6).^[59]^ In contrast, Demchenko and co‐workers leveraged solution phase HPLC‐based automation in 2022, with a lactose building block serving as the core to construct two protected precursors to the trisaccharide HMOs: β3’‐GL **12** and LNTri II **13** in 60 % and 43 % yields, respectively (Figure 6).^[56]^ It is important to stress that the biological effects of these types of structures have been described; for example, β3’‐GL is present in colostrum and has been shown to modulate interleukin‐8 levels and reduce inflammation.^[107]^

## 5 Human Natural Killer‐1

**Figure 7:**
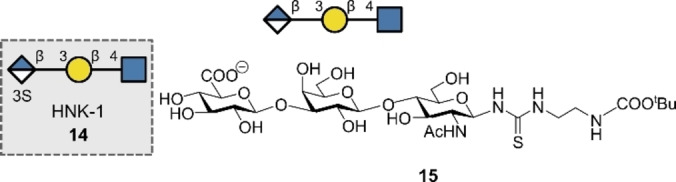
Human natural killer‐1 epitope and a non‐sulfated analogue synthesized with automation.

The Human natural killer‐1 (HNK‐1) epitope **14** is associated with migration and adhesion of cells in the nervous system (Figure 7).^[108]^ The non‐reducing end GlcA of **14** is sulfated, in contrast to the non‐sulfated HNK‐1 epitope **15** prepared in 2019 by Elling, Franzreb and co‐workers.^[61]^ Employing an automated enzyme cascade using an immobilised microfluidic enzyme reactor, the authors produced trisaccharide **15** in 96 % yield. The pharmaceutical relevance of the product is evident from cases of anti‐myelin‐associated glycoprotein (anti‐MAG) neuropathy, where the patient produces anti‐MAG IgM that has been shown to bind the HNK‐1 epitope of MAG.^[109]^ Current treatments include harsh chemotherapy,^[110]^ creating an opportunity to explore alternative approaches: glycomimetic decoys that bind anti‐MAG IgM in place of the natural HNK‐1 are being investigated.^[111]^ It is tempting to speculate that automation could provide an enabling solution by accelerating the generation of libraries of potential HNK‐1 decoys, thereby allowing their binding to anti‐MAG IgM to be rapidly assessed.

## 6 N‐Glycans

Protein glycosylation patterns are post‐translational events with important ramifications in molecular recognition.^[112]^ Functionalised asparagine (Asn) residues give rise to N‐glycosidic bonds, termed N‐glycans,^[113]^ which are implicated in cell signalling and adhesion, including in cancer development.^[114]^ HIV is a notable example of N‐glycosylation mediated infection; a glycoprotein expressed on the envelope of HIV‐1 cells, gp120, can bind host DC‐SIGN via its N‐glycans, which facilitates invasion of host cells.^[39]^ Consequently, the stereocontrolled construction of N‐glycans of gp120 targets is of central importance for HIV vaccine development.^[115]^

**Figure 8:**
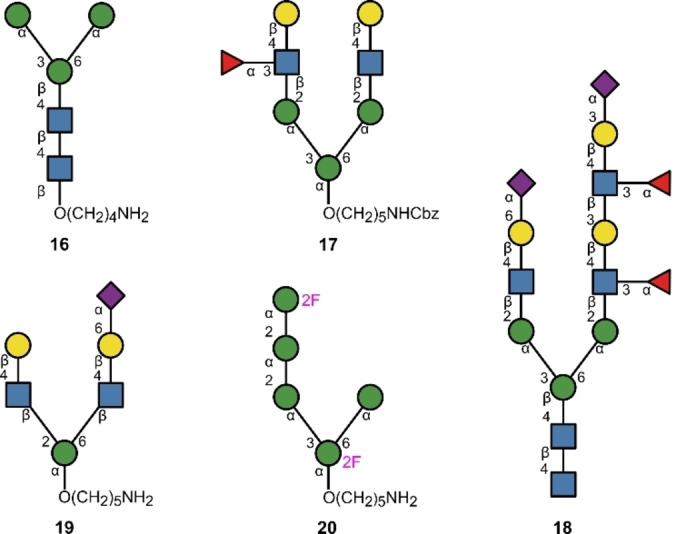
N‐glycan motifs from automated glycan synthesis.

In 2019, Demchenko and co‐workers employed solid phase HPLC‐based automation to synthesise the core N‐glycan pentasaccharide **16** (Figure 8), where the difficult β‐mannose linkage was independently formed in solution and used as a disaccharide building block.^[116]^

In the same year, Seeberger and co‐workers employed solid phase automation to create oligosaccharide donors and acceptors that were used to create an N‐glycan fragment.^[117]^ A trisaccharide donor and a pentasaccharide acceptor (both derived from independent automated syntheses) in a [5+3] solution phase glycosylation gave the protected precursor to octamer **17**; this is a fragment of a biantennary N‐glycan. Boons and co‐workers employed enzymatic automation to furnish a sialylated, fucosylated asymmetric complex glycan 15‐mer **18**.^[59]^ It is interesting to note that the starting molecule was a branched nonamer containing the N‐glycan core that had been isolated from a glycoprotein in egg yolk powder. This was a proof of concept for the platform, showing that even natural glycans could be integrated and extended owing to the protecting group‐free nature of enzymatic synthesis. This is appealing, as the structural complexity that nature is capable of producing can then be used as a starting point to enable rapid access to complex, structurally diverse glycans.

In 2024, Delbianco and co‐workers synthesised a sialylated branched asymmetric N‐glycan fragment **19**,^[118]^ in which solid phase automation was used to generate a precursor with two identical disaccharide branches. The branches were formed sequentially and the last sugar installed bore an Fmoc group, which was ultimately cleaved to allow phosphorylation. Cleavage from the resin and global deprotection furnished the substrate for enzymatic sialylation with CMP‐Neu5Ac. The phosphate masking group was then cleaved with an alkaline phosphatase to yield fully deprotected **19**.

To contribute to the growing interest in glycomimetics,[^11^, ^12^, ^119^, ^120^] Teschers and Gilmour merged solid phase automation with fluorine directed glycosylation^[121]^ to furnish the protected, fluorinated branched N‐glycan pentasaccharide fragment **20**.^[122]^ C2‐fluorination of glycans is a validated strategy to enhance hydrolytic stability,^[123]^ and this modification simultaneously introduces a sensitive NMR handle to facilitate analysis during synthesis as well as in subsequent biological assays.^[84]^ This study served as a proof of concept that fluorine directed glycosylation can be integrated into automated synthesis algorithms to facilitate the synthesis of glycomimetics.

## 7 Chimeras: Glycans, Peptides and Lipids

**Figure 9:**
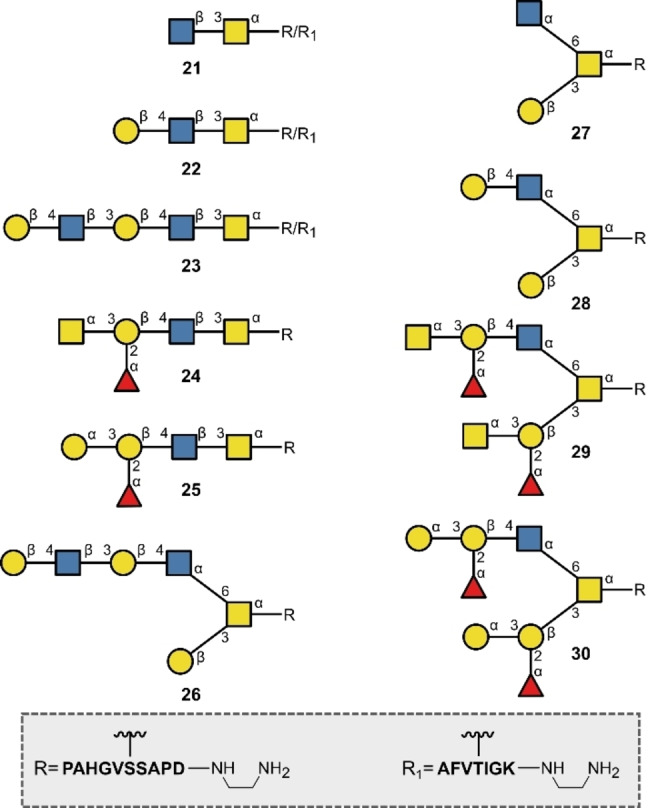
Glycoprotein fragments synthesised with both the protein and glycan units generated by automation.

Glycan patterns on proteins are intimately associated with numerous pathogenic processes including cancer, cystic fibrosis, bacterial and viral infections as well as rheumatoid arthritis.^[124]^ It logically follows that a more detailed understanding of glycoprotein structure and function is necessary to enable growth in the development of glycoprotein‐based therapeutics. Whilst the previous section of this minireview highlighted the importance of N‐glycans by emphasising the structural variation in the glycan portion, it is important to stress that the peptide component is also important and, although non‐trivial, the synergistic automation of peptide‐glycan chimeras is a natural evolution from automated glycan synthesis and SPPS. Methods for glycopeptide synthesis often rely on glycosylated amino acid building blocks that can be extended after SPPS,^[125]^ whereas methods that also automate the glycan extension are rare. In 2020, Wang, Wen and co‐workers disclosed the construction of glycopeptides with SPPS using glycosylated amino acid building blocks in a modified peptide synthesiser.^[62]^ This conceptual paradigm enables the glycan chain to be extended in an automated enzymatic fashion from the corresponding glycosylated amino acids. The glycopeptide PAHGVSSAPD (Figure 9) prepared by this strategy is particularly noteworthy, as it constitutes part of the tandem repeat unit of a cancer‐associated mucin‐type glycopeptide Muc1 (with the glycosylated threonine replaced with serine in this case).^[126]^

Muc1 in cancerous cells is distinct from normal Muc1, whereby incomplete glycosylation results in truncated side chains such as in the venerable Thomsen‐Friedrich, Tn and sialyl‐Tn antigens.^[127]^ As a consequence, Muc1 analogues are promising cancer vaccine candidates and their syntheses have been intensively pursued. It is interesting to note that the HIV related peptide AFVTIGK was synthesised that forms a part of the HIV120gp tandem repeat.^[128]^ These scaffolds (PAHGVSSAPD and AFVTIGK) were subsequently decorated with the glycan epitopes **21**–**30** (Figure 9), which hold great promise in the investigation of host‐pathogen interactions with a more natural presentation of glycans, relative to microarray formats.

**Figure 10:**
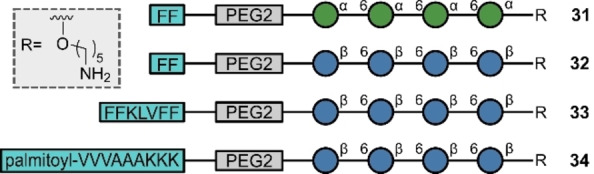
Protein‐glycan chimeras and protein‐glycan‐lipid chimeras synthesized with automation.

In 2023, Seeberger and co‐workers effectively merged solid phase automation and SPPS to enable the synthesis of glycopeptides **31**–**34** with the peptide chain at the non‐reducing end of the glycan (Figure 10).^[129]^ The terminal sugar on the glycan bore a protected amino group that was used as a starting point for the SPPS. This approach thus provides an expansive opportunity to conjugate the glycans/glycopeptides to lipids and steroids, thereby demonstrating the potential of automation in the production of complex biomolecules.

## 8 Multivalency

**Figure 11:**
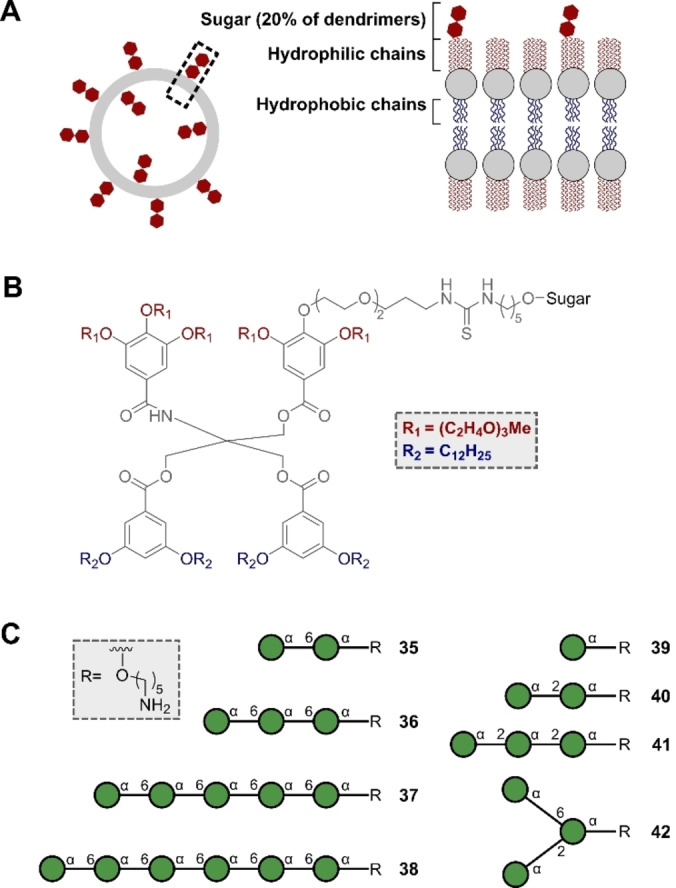
A) Simplified depiction of a dendrimersome. B) Chemical structure of dendrimers. C) Structure of the glycans bound to the dendrimers.

Protein‐glycan binding is often multivalent,^[130]^ which is challenging to replicate using glycans immobilised on slides in an ill‐defined arrangement.^[131]^ Array printing techniques have advanced to allow for well‐defined glycan spacing and densities, helping to unmask the influence of multivalency in glycan binding. In 2020, Loeffler and co‐workers reported a study in which spatially defined arrays were designed to investigate multivalency; of the structures examined, an α‐1,6‐dimannoside was derived from automated synthesis.^[132]^ The authors investigated the binding of the glycans (bound to peptides on a surface) to concanavalin A (Con A). Con A is a mannose‐binding plant lectin with a monosaccharide binding pocket. The specificity of this site can be summarised by the Goldstein rules, which state that an equatorial 3‐OH and 4‐OH as well as a free primary alcohol at C6 are required.^[133]^ Con A is known to bind to the surface antigens of pathogens including *E. coli*, making it a highly pertinent lectin for analysis.^[134]^ In this study, the authors noted that an exponential increase in binding was observed with a linear increase in the number of monosaccharide units in the glycans on the array; this is fully in‐line with multivalent binding. However, despite the advances conferred by using spatially‐defined glycan arrays, achieving a natural presentation format of the glycans remains challenging. An emerging strategy to reconciling this difference is the use of glycosylated membrane mimics such as Janus glycodendrimers.^[135]^ In 2020, Klein, Seeberger, Percec and co‐workers investigated the synthesis of Janus glycodendrimers, including the mannose dendrimers **35**–**42** (Figure 11A), using a solid phase automated platform to synthesise the glycans.^[136]^ In this study, an alternative reaction to the commonly used copper‐catalysed azide‐alkyne cycloaddition (CuAAC) was disclosed to couple the Janus dendrimers (Figure 11B) to the glycans (Figure 11C): an isothiocyanate‐amine click reaction that utilised the amine‐containing linker of the automation‐derived glycans. The glycodendrimers self‐assembled into glycodendrimersomes that were used to investigate agglutination to Con A. The authors investigated the influence of glycan length on binding and discovered that a mannose hexamer showed the most effective binding when compared with mono‐, di‐ and trisaccharides. This contribution to the field of Janus dendrimer‐based membrane mimics serves as a valuable proof of concept that (with the isothiocyanate‐amine click reaction) automation has the potential to greatly expedite the generation of glycosylated membrane mimics. In addition to binding studies, the interest in leveraging Janus dendrimersomes to encapsulate drugs will now be able to benefit from automation, a rapid synthesis platform to accelerate the preparation of compound libraries.^[137]^

Nanoparticles are also appealing in emulating the multivalent display of glycans on cell surfaces and in 2022, Seeberger and co‐workers reported gold nanoparticles decorated multivalently with non‐immunogenic α‐(1‐6)‐oligomannans.^[138]^ A collection of candidates ranging from a linear disaccharide up to tetrasaccharides was made in an automated fashion; these compounds were shown to activate the innate immune response but not the complement system.

## 9 TMG‐Chitotriomycin

**Figure 12:**
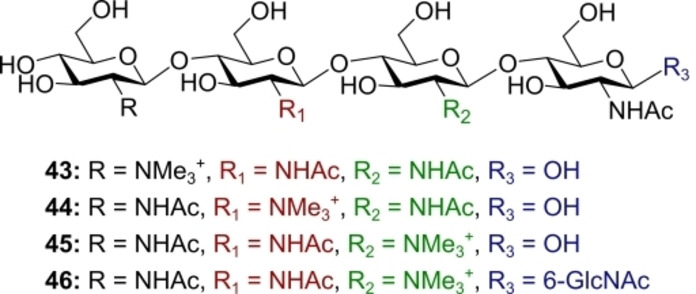
Structures of TMG‐chitotriomycin and analogues prepared by automated electrochemical glycan synthesis.

TMG‐chitotriomycin **43** (Figure 12), which contains a conspicuous *N*,*N*,*N*‐trimethyl‐d‐glucosamine motif, is a GlcNAcase inhibitor that shows selectivity towards insects and fungi but not plants and mammals.^[139]^ Interestingly, inhibitory activity is lost when using only the trimethylammonium‐containing monosaccharide but it is restored when linked to the reducing end GlcNAc trimer. Accumulating mechanistic information and delineating structure‐activity relationships is of translational relevance, and thus analogue synthesis is highly enabling.[^140^, ^141^, ^142^] Electrochemical automated glycan synthesis had been previously leveraged to prepare TMG‐chitotriomycin **43** by Nokami, Itoh and co‐workers.^[143]^ In 2023, Nokami and co‐workers employed electrochemical automation to enable the synthesis of a small library (Figure 12), in which the position of the trimethylammonium group was varied: 2‐TMG chitotriomycin **44**, 3‐TMG chitotriomycin **45** and also 3‐TMG‐NAG_5_ **46**, with an extra GlcNAc residue at the reducing end.^[144]^ The synthesis of TMG‐chitotriomycin further underscores the importance of total synthesis campaigns in structural characterisation. The initial configuration of the non‐reducing end linkage in TMG chitotriomycin was proposed to be α‐configured^[139]^ and was reassigned as β‐configured.^[145]^

## 10 Bacterial Glycans

**Figure 13:**
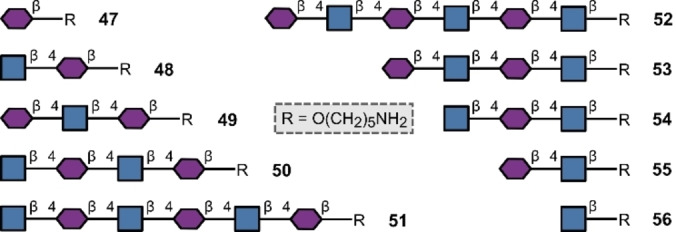
Peptidoglycan fragments prepared with automated glycan synthesis.

The translational importance of bacterial LPS O‐antigens in biomedicine[^7^, ^8^] continues to be a powerful driver of innovation in the development of enabling synthetic methods. Automation remains in the vanguard of approaches to prepare compounds for biological applications with vaccine leads for *Streptococcus pneumoniae* serving as powerful exemplars.[^40^, ^146^] Importantly, other components of the bacterial cell wall are now attracting considerable interest from the synthesis community. Pertinent to this minireview is peptidoglycan, a mesh‐like structure of linear glycans (β‐1,4‐linked GlcNAc and MurNAc) and cross‐linking peptides that is found in most bacteria, including *Mycobacterium tuberculosis*.^[147]^ This target is structurally vital for the bacterium and counteracts osmotic pressure which helps the cell to retain its shape: it has also been reported to activate the innate immune system. Motivated by the dearth of structural information regarding which epitopes regulate host‐pathogen interactions, Loeffler and co‐workers prepared fragments of the bacterial peptidoglycan backbone utilising a muramic acid building block to synthesise 10 linear glycans **47**–**56** ranging from mono‐ to hexa‐saccharides with solid phase automation (Figure 13).^[148]^

**Figure 14:**
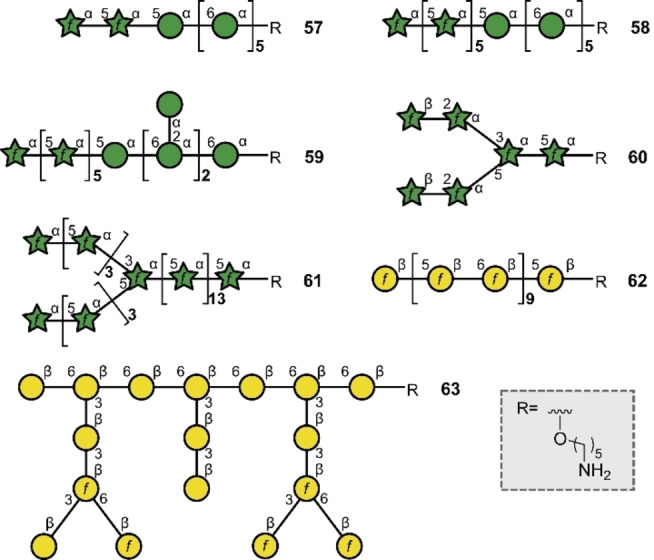
Bacterial glycans prepared by automation.

These structures provide a valuable foundation from which to probe the binding of peptidoglycan to host receptors and identify potential targets for drug development. The mycobacterial cell wall, aside from peptidoglycan, also contains arabinogalactan and lipoarabinomannan which are appealing candidates for drug discovery campaigns. Whilst many established tuberculosis drugs operate by inhibiting mycolic acid production in the cell wall, routes to access arabinogalactan, lipoarabinomannan and peptidoglycan have been intensively pursued.^[149]^ In 2019, Seeberger and co‐workers produced three structures (**57**–**59**) (Figure 14) by solid phase automation, based on portions of the core arabinomannans found in *M. tuberculosis*.^[150]^ A significant advance in this area was the generation of a 6‐mer **60**, containing two β‐arabinofuranose linkages and 23‐mer arabinofuranoside **61**.^[151]^ The protected 23‐mer was generated in an impressive 13 % yield over 57 steps, further underscoring the enabling nature of automation. Furthermore, aided by the fact that furanoses are not common in humans, arabinofuranoses have already shown promise as vaccine leads for *M. tuberculosis*.^[152]^ Although non‐bacterium‐derived, it is relevant to highlight a related arabinogalactan isolated from *Carthamus tinctorius* (safflower), named HH1‐1, which had previously been shown to have immune modulating properties.^[153]^ Having produced linear 20‐mer galactofuranoside **62** in 2021,^[154]^ Seeberger and co‐workers forged the branched arabinogalactan heptadecasaccharide **63** repeat unit of HH1‐1 using solid‐phase automation two years later (Figure 14).^[155]^ The therapeutic opportunities of distilling the minimal epitopes required to elicit immune modulation is evident from the study by Luo and co‐workers with other safflower polysaccharides.^[156]^

## 11 Sulfated Glycans

**Figure 15:**
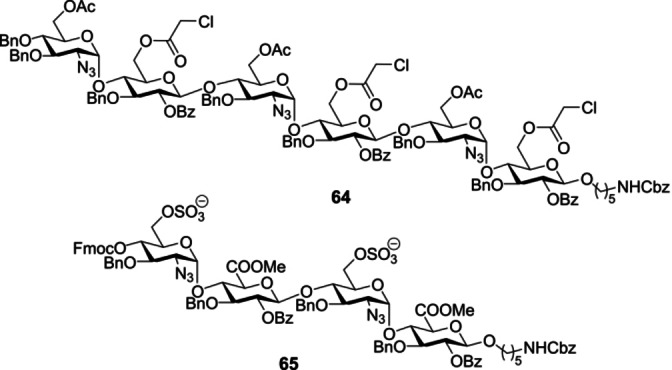
Precursors to heparan sulfate made with automation.

The antithrombotic properties of heparin render it a compelling exemplar of the societal relevance of sulfated glycans. In addition to their structural diversity, sulfation patterns are a powerful modulator of function in glycobiology.^[157]^ A recent study by Hsieh‐Wilson and co‐workers has shown that interrogating this additional level of complexity, and placing function on a structural footing, can be expedited through the development of an efficient synthesis platform merged with glycoarray technology.^[158]^ Utilising a traceless fluorous tagging strategy, the authors successfully generated 64 discrete heparin sulfate analogues, in which all possible 2‐*O*‐, 6‐*O*‐ and *N*‐constellations in a model tetrasaccharide (GlcN–IdoA‐GlcN‐IdoA) were generated. Automated approaches include a study by Fascione and co‐workers who disclosed an automated synthesis of a heparan sulfate precursor on solid phase.^[159]^ Hexasaccharide **64** was assembled in 6 hours in 30 % yield and required only flash chromatography for purification (Figure 15). This approach leveraged a disaccharide GlcN‐α4‐Glc building block, in which the challenging α‐linkage was pre‐installed. The most recent solid phase strategy employed by Miller and co‐workers centred upon the disaccharide GlcN‐α4‐GlcAto enable the protected heparan sulfate precursor **65** in 3 % overall yield (Figure 15).^[160]^ The authors specifically validated the use of a GlcA‐containing building block to mitigate the need for post‐automation oxidation required when using Glc building blocks.

**Figure 16:**
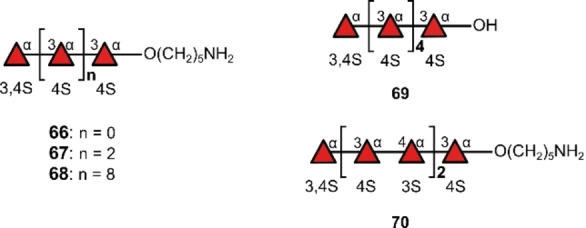
Sulfated fucoidan fragments made by automated glycan synthesis.

Aside from anticoagulant activity, heparan sulfate is of great interest in the study of Alzheimer's disease, where it is thought to be involved in β‐amyloid protein (Aβ) forming plaques and tau protein forming tangles.^[37]^ Rhamnan sulfates also possess anticoagulant activity^[161]^ and fragments have been synthesised using solution phase automation by Pohl and co‐workers.^[162]^ Fucoidans are another important class of sulfated polysaccharides that contain fucose and can act as antiviral,^[163]^ anti‐tumour^[164]^ and anticoagulation agents.^[165]^ Seeberger and co‐workers reported the solid phase automation of a collection of sulfated algal fucoidans **66**–**70**, including a 10‐mer (Figure 16).^[166]^ Merging automated synthesis with microarray technology enabled the authors to investigate antibody binding and to characterise various hydrolases.

The effective characterisation of hydrolases is vital in a therapeutic context as larger molecular weight fucoidans are required to enable anticoagulant activity compared to other sulfated structures: a compelling example is that thrombin inhibition requires a minimum of a 26‐mer for heparin^[165]^ but a 408‐mer for fucoidan.^[167]^ These structures would be beyond the comfortable reach of current automation technology. However, by characterising hydrolases on smaller standards, these enzymes could conceivably be harnessed for the more controlled cleavage of natural fucoidans to yield therapeutic candidates.

**Figure 17:**
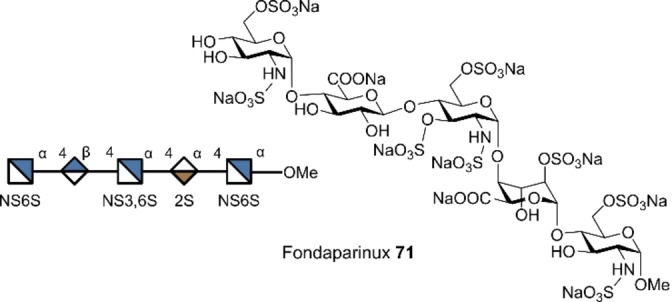
Structure of the anticoagulant fondaparinux (Arixtra®).

Fondaparinux **71**, used as the sodium salt (Figure 17), is a synthetic anticoagulant that mimics the binding site of heparin to antithrombin III, whilst reducing the risk of heparin‐induced thrombocytopenia and boasting a longer half‐life than heparin.^[168]^ Efforts to streamline the synthesis of this important sulfated glycan have been ongoing since its discovery.[^169^, ^170^] In 2022, Ye and co‐workers synthesised a protected precursor of fondaparinux on gram scale in 62 % yield using a multiplicative paradigm.^[43]^ This expedited access to the drug precursor has the potential to lower production costs and improve accessibility.

## 12 Future Directions

Innovative strategies to enable the automated assembly of complex glycostructures are vital in reconciling the importance of therapeutic glycans with the challenges associated with their synthesis. The absence of bioinspired amplification blueprints distinguishes the stereocontrolled construction of carbohydrates from the linear union of peptides and nucleotides. However, these fields share the common objective of seeking to establish synthesis algorithms that allow even the most exotic biopolymers to be prepared with iterative precision and in a robust and reproducible manner. The expansiveness of glycospace^[18]^ continues to reshape the preparative carbohydrate chemistry landscape and these horizons will continue to expand as enabling automation platforms are more widely adopted by practitioners. Technological advances such as rapid temperature regulation^[75]^ and integrated purification platforms^[171]^ will enhance the appeal of the conceptual paradigm by further streamlining challenging syntheses. The ability to effortlessly unify automated glycan synthesis with other biomolecule assembly platforms will create a modularity that further increase the appeal and render the technology accessible to non‐specialists.

Whilst surveying recent advances in the automated synthesis of therapeutically relevant glycans, the *status quo* in both enzymatic glycosylation (using unprotected donors and enzymes) and chemical glycosylation (using protected donors and activators) has become apparent. Whereas enzymatic methods mitigate the need for protecting group regimes and benefit from excellent levels of (α/β)‐diastereoselectivity, they are frequently characterised by long reaction times.^[59]^ In stark contrast, non‐enzymatic methods profit from shorter reaction times but require judicious protecting group patterns to orchestrate selectivity. Regulating chemical sialylation,^[172]^ or generating 1,2‐*cis* linkages^[173]^ are two illustrative example of the contemporary challenges that require effective solutions.

It is envisaged that enzymatic automated glycan synthesis will see rapid growth as the cost of enzymes decrease, and the feasibility of higher enzyme loadings increases: this will be accompanied by a reduction in synthesis times. An interesting observation that derived from writing this minireview was the unexpectedly high number of reports in which the target glycans were not globally deprotected. This may be attributable to: i) automated glycan synthesis being a relatively new technology and the conscious report of a proof of concept, or; ii) this observation reflects the intractable challenges associated with fully deprotecting and purifying glycans. These issues will become centre stage as the trend to generate therapeutic glycans via automation continues. Whilst the advantages and disadvantages of individual approaches will continue to stimulate discussion and drive innovation, it is indisputable that this enabling technology has accelerated glycospace exploration at an unparalleled rate. The complexity and diversity of therapeutic glycans synthesised by automation over the past five years is a compelling indication that this powerful technology will continue to expedite discovery.

## Conflict of Interests

The authors declare no conflict of interest.

13

## Biographical Information

*James Suri studied chemistry at the University of St Andrews, spending one year as a computational chemist at Optibrium Ltd., developing machine learning models of drug metabolism. He completed his master‘s (M.Chem. (Hons), 2021), under the supervision of Dr. Christopher Lancefield, investigating the degradation of lignin by fungi. He then moved to the University of Münster to pursue his doctoral studies in the group of Prof. Dr. Ryan Gilmour, where his research concerns the automated synthesis of fluorinated carbohydrates with therapeutic potential*.

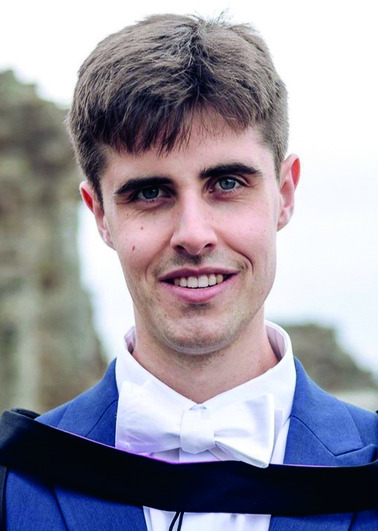

## Biographical Information

*Ryan Gilmour was born in Ayrshire, Scotland (1980) and studied at the Universities of St Andrews (M.Chem. (Hons), 2002) and Cambridge (PhD, 2006). Following research stays at the Max Planck Institute (Mühlheim/Ruhr), and the ETH Zurich, he was appointed to the ETH Zurich in 2008. In 2012, he moved to the University of Münster where he is Chair of Organic Chemistry and CiMIC Professor of Chemical biology. Gilmour is a Fellow of the Royal Society of Chemistry (2015), a Corresponding Fellow of the Royal Society of Edinburgh (2021) and a Fellow of the European Academy of Sciences (2023)*.

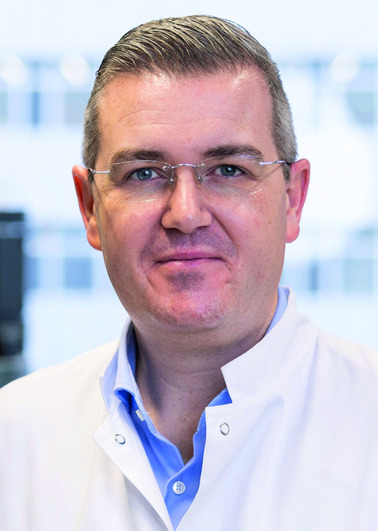

## Data Availability

The data that support the findings of this study are available from the corresponding author upon reasonable request.
